# Supplementary material for: NEDD4 controls spermatogonial stem cell homeostasis and stress response by regulating messenger ribonucleoprotein complexes
Source: Nat Commun. 2017 Jun 6;8:15662. doi: 10.1038/ncomms15662 (PMC5494183; doi:10.1038/ncomms15662)
Supplement: Supplementary Information — Supplementary Figures, Supplementary Tables, Supplementary Methods, Supplementary References. [file ncomms15662-s1.pdf]

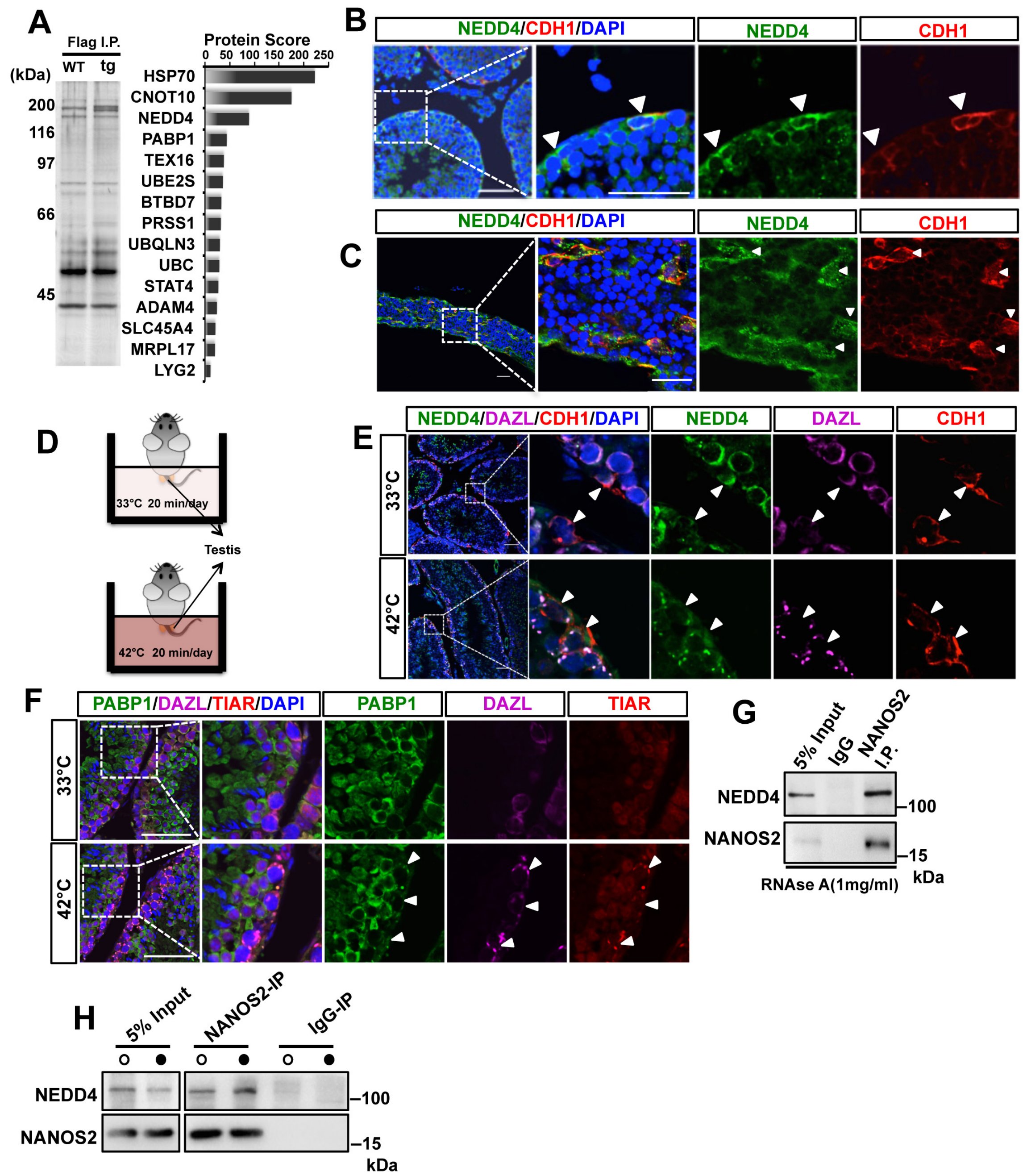

Supplementary figure 1. Heat stress induces SGs in spermatogonia. Related to Figure 1.

(A) NANOS2 interacting proteins were identified by mass spectrometry/mass spectrometry (MS/MS) in male gonads. Positive control of NANOS2 interacting proteins, such as CNOT1 [1, 2] and PABP1 [3], were identified. Another NANOS2 binding candidate NEDD4 was identified. Tg indicates a mouse line that expresses Flag-tagged NANOS2 under the control of the *Nanos2* enhancer [1, 2].

(B-C) Representative staining patterns of sections (B) and whole mount (C) of WT (wild-type) adult testes (12 weeks) are shown. Immunostaining was performed with anti-NEDD4 and anti-CDH1 antibody. Enriched NEDD4 was observed in $CDH1^+$ SPCs. Scale bars = 50μm.

(D) Illustration shows the method of the heat shock treatment for testis *in vivo*.

(E, F) Representative staining patterns of heat shock-treated testes. Frozen sections were stained for NEDD4, DAZL and CDH1. Heat shock-induced changes in the subcellular distribution of DAZL and NEDD4 in $CDH1^+$ SPCs (E). PABP1 was also recruited to SGs (marked by DAZL and TIAR) in cells indicated by arrowheads (F). Scale bars = 50μm.

(G) GSC lysates were treated with 1 mg/ml RNAase A and then immunoprecipitation was performed with a NANOS2 antibody. Results indicated an RNA-independent interaction between NANOS2 and NEDD4.

(H) Western blots of a co-IP experiment with an anti-NANOS2 antibody and protein lysates from GSCs incubated at 33°C (open circles) and 42°C (filled circles) for 20 min.

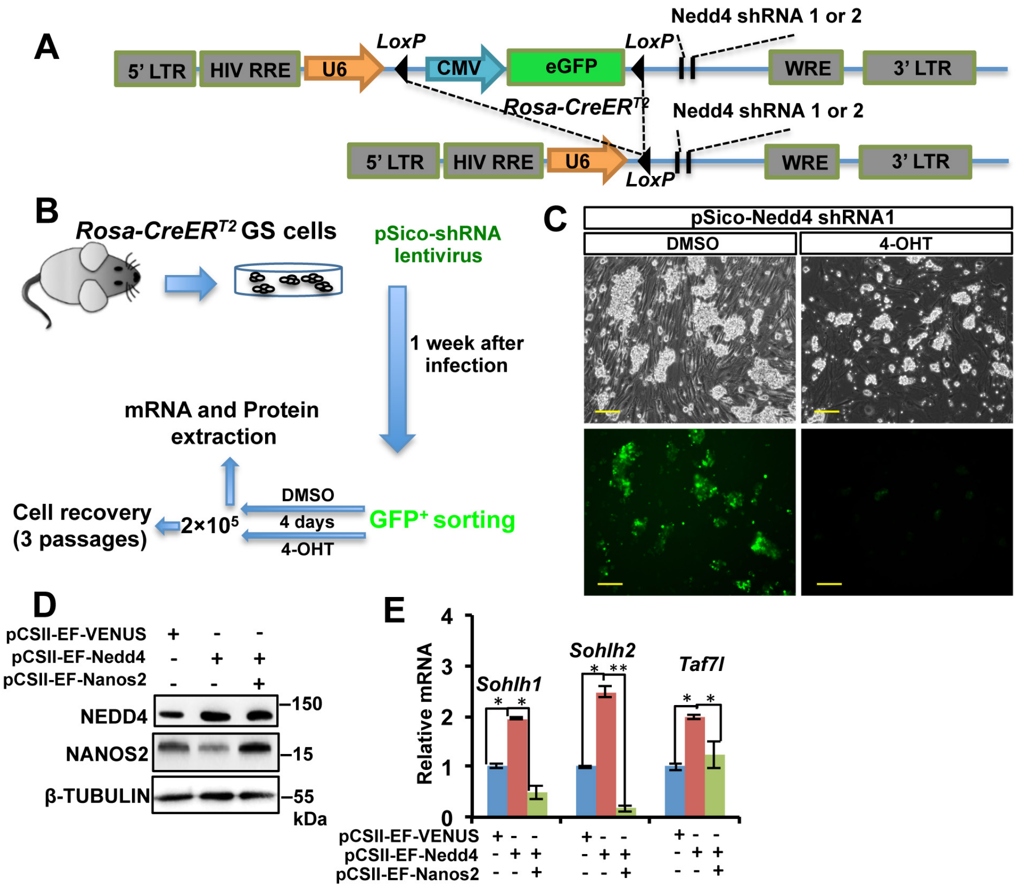

Supplementary figure 2. Function of NEDD4 in GSCs. Related to Figure 2.

(A) A lentiviral plasmid construct used for knockdown of NEDD4. *Nedd4* shRNAs (1 and 2) were inserted in pSICO plasmids. After recombination via Cre, *Nedd4* shRNA is induced.

(B) Experimental procedure used for the establishment of Nedd4 conditional knockdown GSCs (*Nedd4*-cKD GSCs).

(C) Representative pictures of GSCs containing pSico-*Nedd4* shRNA1 treated with DMSO or 4-OHT for 1 week, respectively. Loss of GFP indicates Cre recombination. Cell growth was significantly repressed by the *Nedd4* shRNA induction. Scale bars = 50μm.

(D) GSCs were infected with lentivirus vectors that express VENUS (control), NEDD4 and NANOS2 as indicated for 4 days. WB was performed with lysates.

(E) qRT-PCR analysis of key differentiation genes in (D). GSCs infected with the VENUS virus were set as 1, and the relative levels in other treatments are shown as mean ± SD, *n=3*. $^{*}p<0.05$, $^{**}p<0.01$, *t* test.

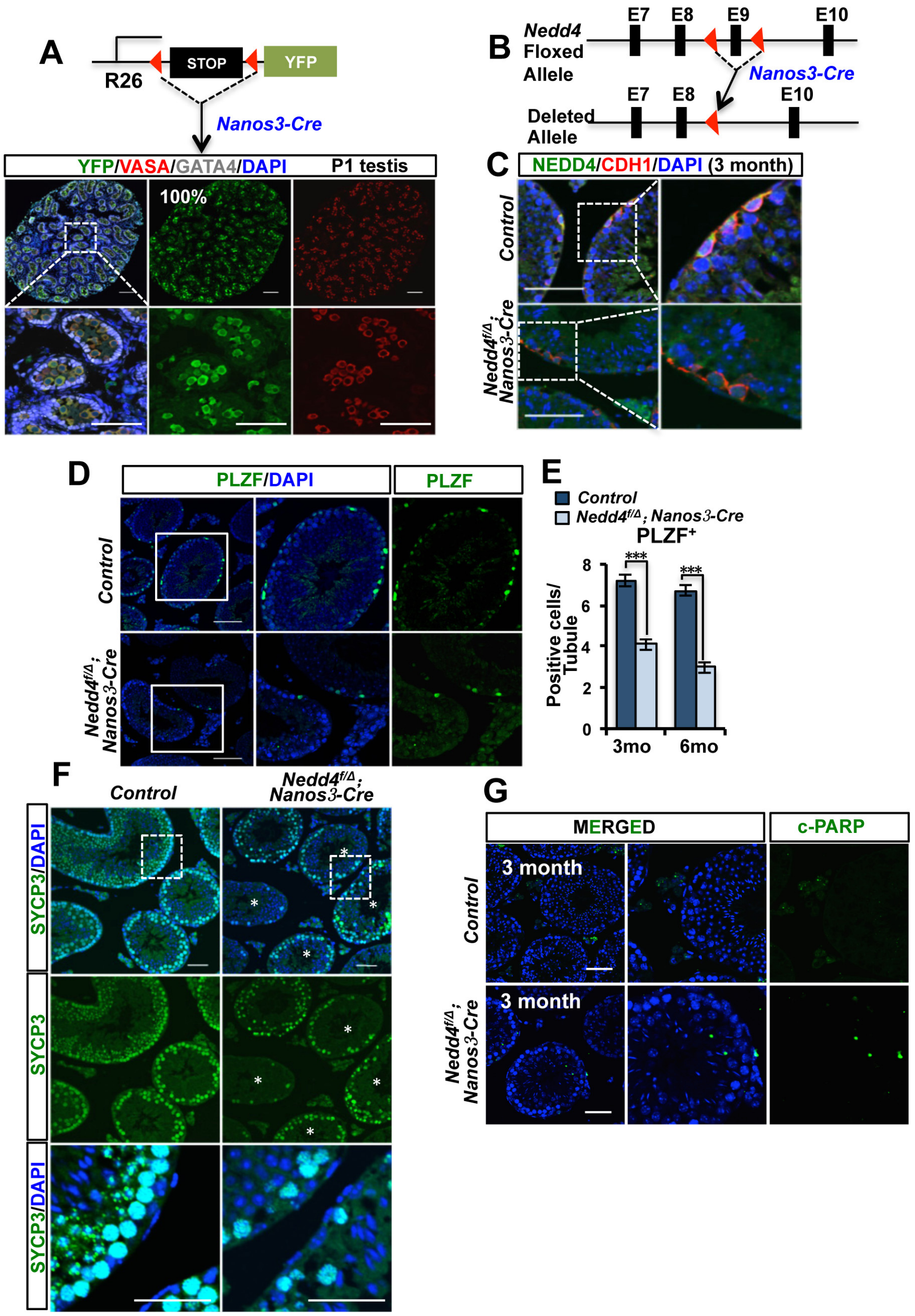

Supplementary figure 3. Defective spermatogenesis in *Nedd4* cKO mice. Related to Figure 3.

(A) *Nanos3-Cre* efficiency was examined by crossing with the Rosa-YFP reporter mice. Almost 100% of germ cells show YFP signals demonstrating higher *Nanos3 Cre* efficiency. Scale bar = 50μm.

(B) *Nedd4* cKO strategy. (C) Immunostaining was performed with anti-NEDD4 and anti-CDH1 antibodies in control and *Nedd4* cKO mice at 3 months. Deletion of *Nedd4* was observed in

CDH1$^+$ SPCs by *Nanos3-Cre*. Scale bar = 50μm.

(D, E) Immunostaining for the SPC marker PLZF showed that PLZF$^+$ cells were significantly reduced in *Nedd4* cKO testes. ***$p<0.001$, *t* test. Scale bar = 50μm.

(F) Representative staining showed that the SYCP3$^+$ meiotic cells were reduced in *Nedd4* cKO testis as compared with the control. Asterisks indicate defective tubules. Scale bars = 50μm.

(G Immunostaining of c-PARP demonstrated no apparent increase of apoptosis in 3-month testes in the absence of *Nedd4*. Scale bars = 50μm.

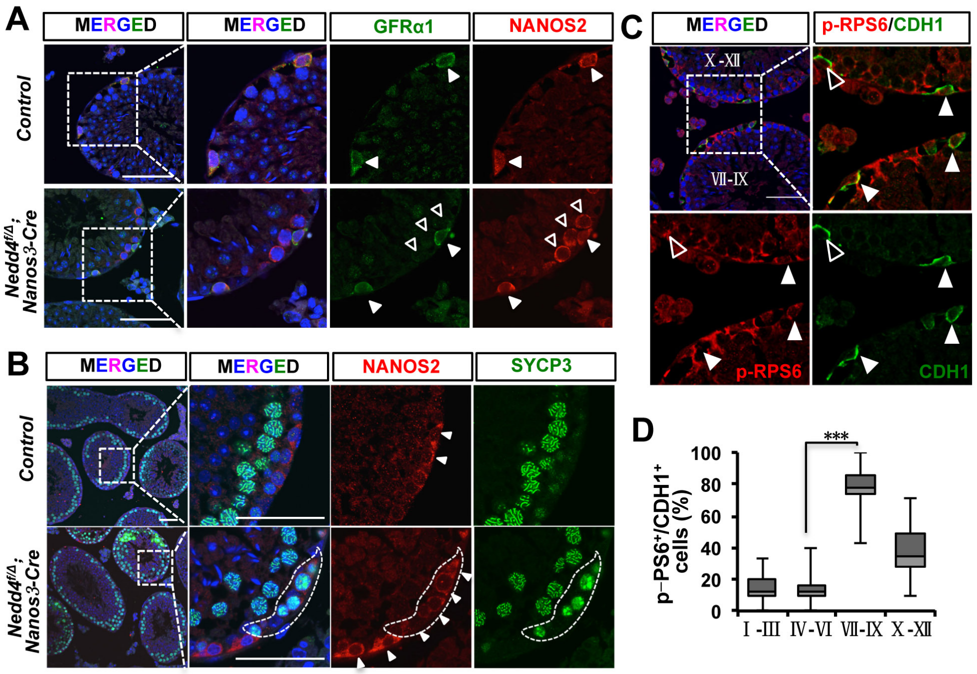

Supplementary figure 4. Spermatogonial defects in *Nedd4* cKO mice. Related to Figure 4.

(A) In control testis (upper panel), GFRα1 (green) and NANOS2 (red) are always co-localized; however, in the *Nedd4* cKO testes, the NANOS2^{+} population was detected even in GFRα1-negative cells (open arrowheads). Scale bars = 50μm.

(B) Representative staining of 3 month old testes showing NANOS2 protein was stabilized even in the early SYCP3^{+} meiotic population in *Nedd4* cKO testis as indicated by arrowheads in dotted circles. Scale bar = 50μm.

(C-D) Co-staining of p-RPS6 with CDH1 (B) respectively in 3-month-old WT mouse testes. Open arrowheads indicate p-RPS6-negative spermatogonia. Filled arrowheads indicate p-RPS6-positive spermatogonia. Statistical analysis indicated that most CDH1^{+} SPCs were p-RPS6^{+} in RA rich stages (C), ****p<0.001*, *t* test. Scale bar = 50μm.

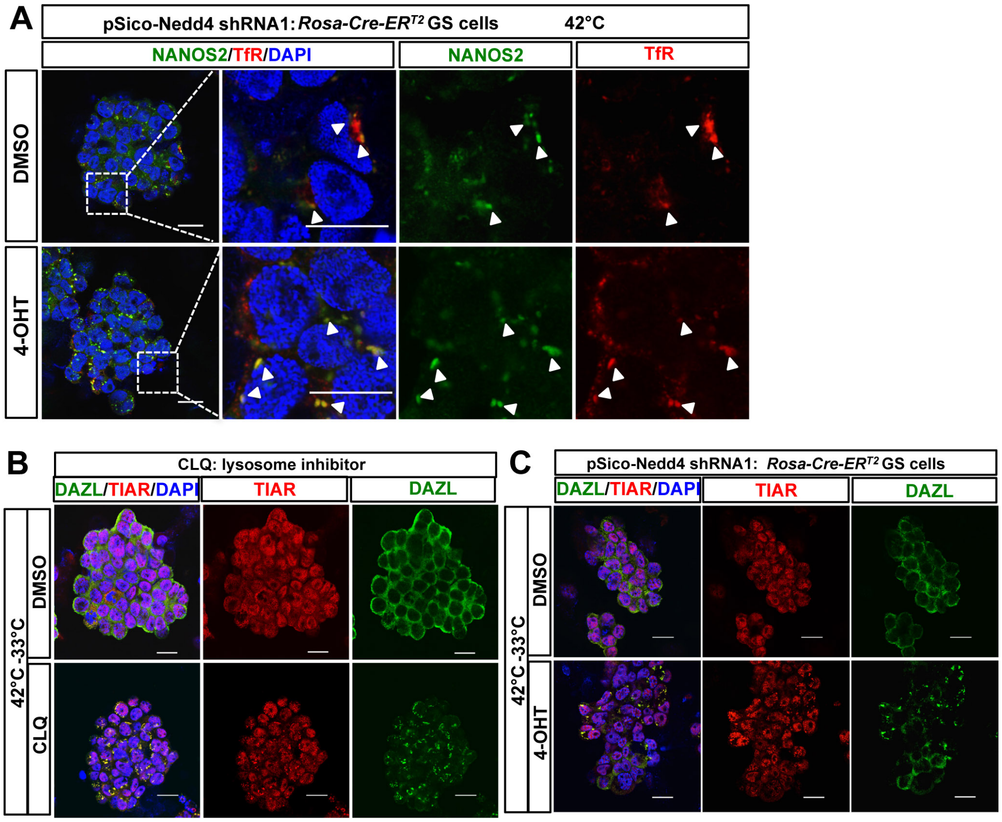

Supplementary figure 5. NEDD4 is required for SG clearance after heat shock in GSCs. Related to Figure 5.

(A) Representative pictures showing staining patterns of NANOS2 and TfR (SG marker) in *Nedd4* cKD GSCs treated with DMSO or 4-OHT for 96 hours, then treated with heat for 20 min. NANOS2 was aggregated in SGs in both control and *Nedd4* knockdown cells; many foci also co-localized with TfR, as indicated by arrowheads. Scale bars = 10 μm.

(B-C) SG clearance was inhibited in both CLQ-treated (B) and *Nedd4* knockdown GSCs (C). GSCs were placed in heat shock conditions (42°C) for 20 min, and then recovered at 33°C for another 3 hours. Scale bars = 10 μm.

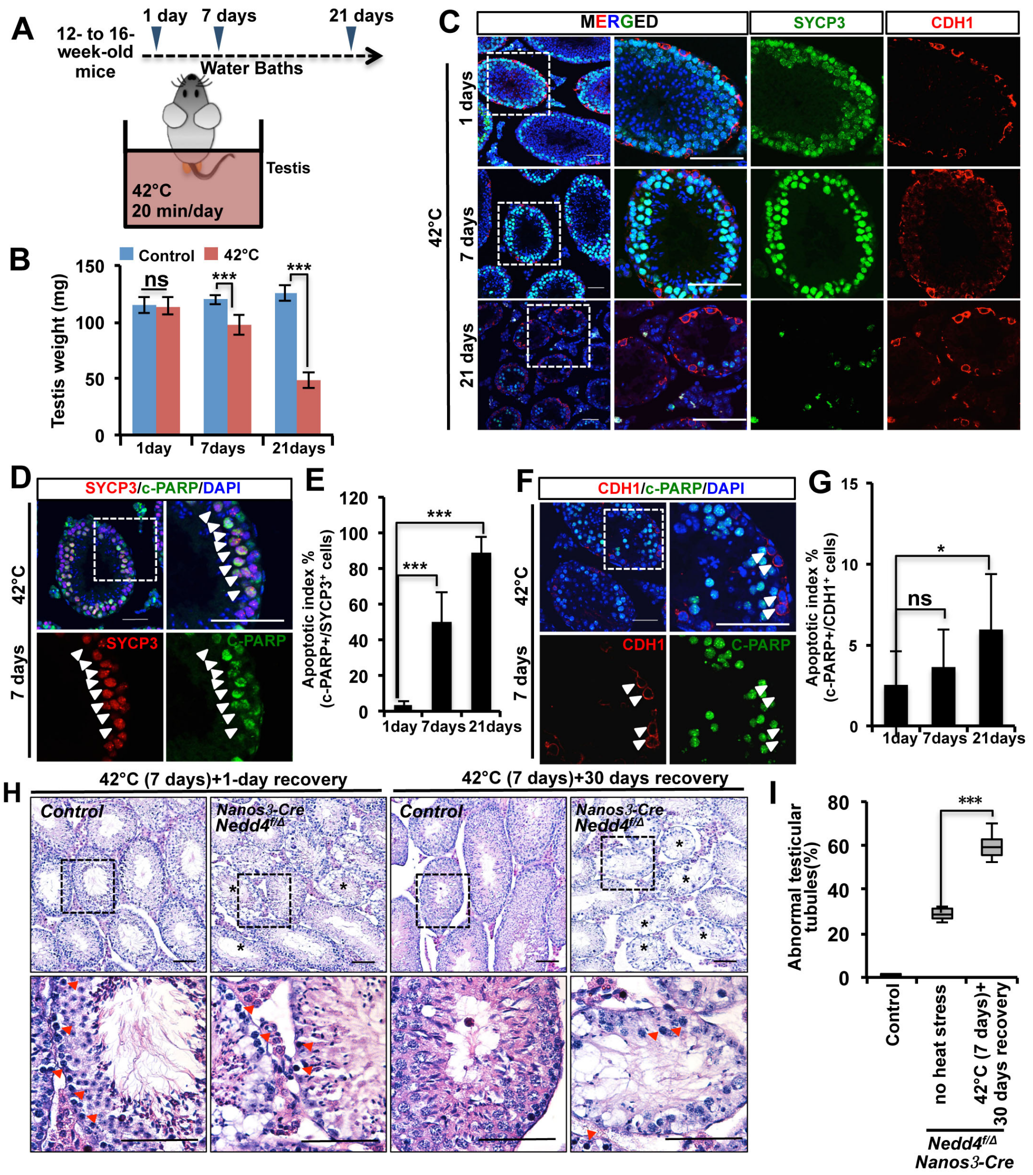

Supplementary figure 6. Effects of heat stress on mouse spermatogenesis. Related to Figure 6.

(A) Illustration shows procedures used for examination of time-dependent heat shock effects in WT adult mice.

(B) Statistical analysis demonstrated the testis weight was significantly reduced after 7-day and 21-day constitutive heat shock. ****p<0.001, t* test. (C) Representative pictures showing the effects of different heat shock times on SYCP3$^+$ meiotic cells and CDH1$^+$ SPCs. SYCP3$^+$ cells were significantly reduced by heat stress treatment. CDH1$^+$ SPCs remained stable in the heat stress conditions. Scale bars = 50 μm

(D-G) Heat induced apoptosis in CDH1$^+$ SPCs and SYCP3$^+$ meiotic cells. After 7-day heat stress, about half of SYCP3$^+$ meiotic cells were c-PARP positive (D). However, no significant c-PARP$^+$ cells were induced in CDH1$^+$ SPCs (F). The proportion of apoptotic cells in the SYCP3$^+$(E) and CDH1$^+$ (G) populations is shown as the mean ± SD. *$p<0.05$, ****$p<0.001$*, *t* test.

(H) Representative H&E staining showing a greater loss of germ cells in *Nedd4* cKO testes by 30-day recovery from heat stress, whereas the control testes recovered normal spermatogenesis. Red arrowheads indicate apoptotic cells with deep hematoxylin staining. Scale bars = 50 μm. (I) Comparison of abnormal testicular tubules (indicated by asterisks) in Nedd4-cKO mice after 30-day recovery, tubules with significantly reduced germ cells were counted and shown as mean ± SD.. ***$p<0.001$, *t* test.

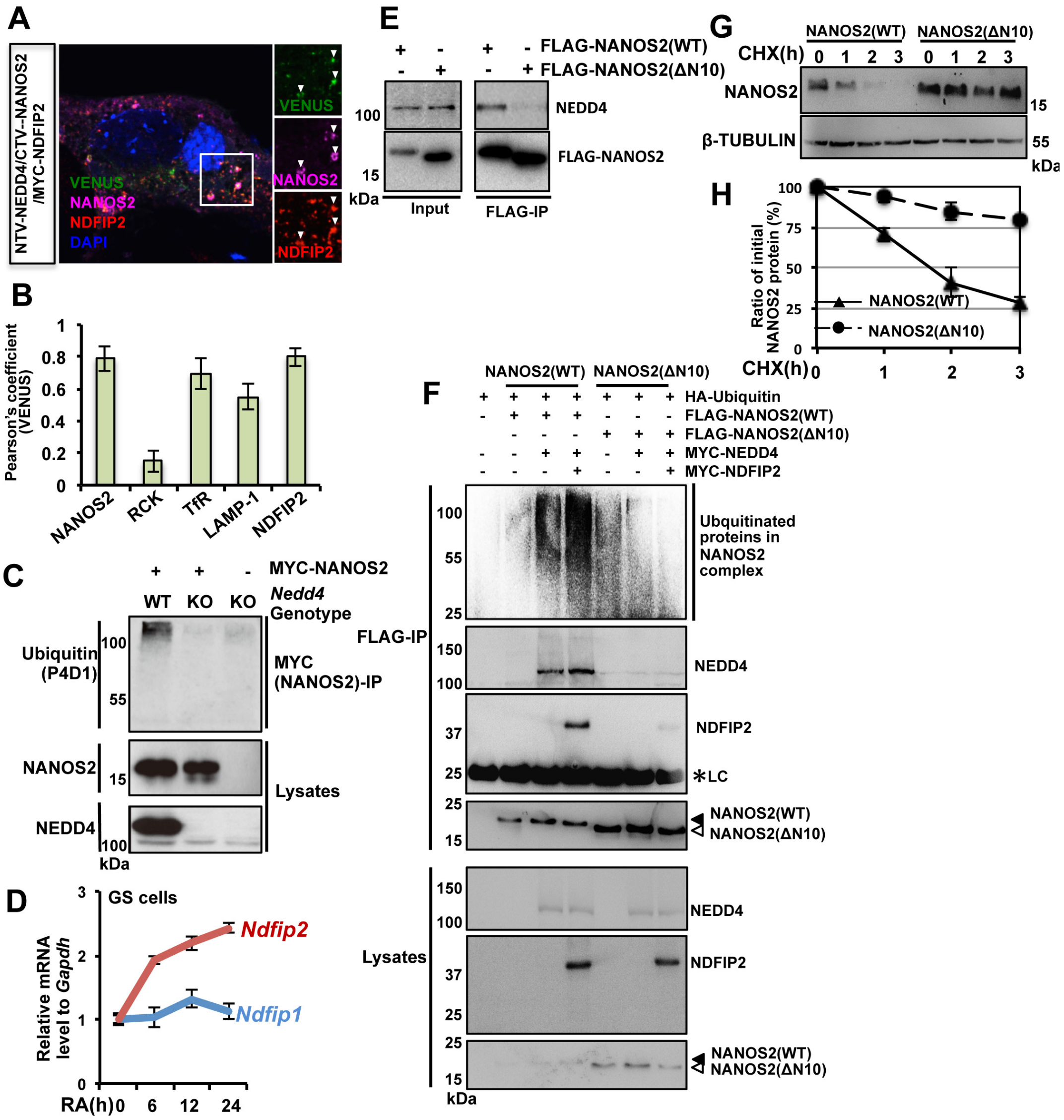

Supplementary figure 7. N-terminal proline-rich region of NANOS2 is critical for its binding to the NEDD4/NDFIP2 complex and ubiquitination by NEDD4. Related to Figure 7.

(A) Representative images showing Venus signals from NEDD4/WT-NANOS2 complex co-localized with MYC-tagged NDFIP2.

(B) Co-localization of VENUS (NEDD4 and NANOS2 interaction) with markers shown in Figure 3 (D-H) was characterized by Pearson's coefficient.

(C) MYC-NANOS2 was expressed in both wild-type and *Nedd4*-KO MEF cells. MYC-IP-western analysis showed that the loss of endogenous NEDD4 reduced NANOS2 protein ubiquitination.

(D) RA induced *Ndfip2* expression in GSCs in a time-dependent manner.

(E) FLAG-tagged NANOS2 (WT: wild type; ΔN10: N terminal deletion) and MYC-NEDD4 plasmids were co-transfected in HEK 293T cells, and then FLAG-tagged NANOS2 was immunoprecipitated with the FLAG antibody.

(F) Western blot analysis of 293T cells co-transfected with plasmids expressing HA-tagged ubiquitin, FLAG-tagged NANOS2 (WT or ΔN10), and MYC-tagged Ndfip2 and NEDD4.

Immunoprecipitation was performed with an anti-FLAG (NANOS2) antibody. WT, wild type NANOS2; ΔN10, N-terminal deleted NANOS2.

(G, H) 293T cells were transfected with FLAG-tagged WT or ΔN10 NANOS2 (1 μg each) for 24 hr, and then CHX (20nM) chase was performed for the indicated time points. Samples were collected for western blotting, and the intensity of FLAG-NANOS2 protein was measured as shown in (H). WT, wild-type NANOS2; ΔN10, N-terminal deleted NANOS2.

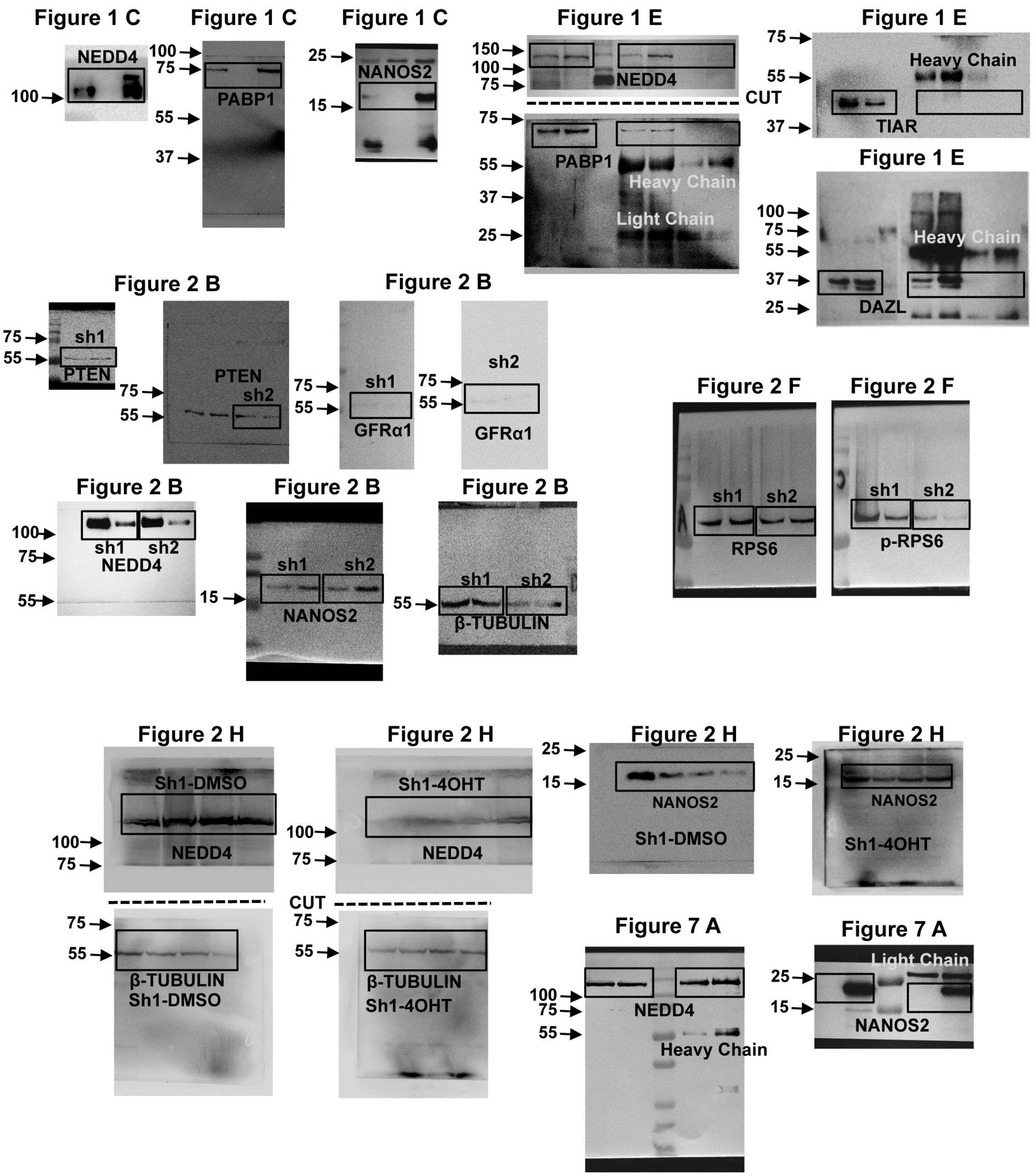
Figure 1 C
NEDD4
100
Figure 1 C
100
75
PABP1
55
37
Figure 1 C
25
NANOS2
15
Figure 1 E
150
100
75
NEDD4
CUT
75
PABP1
55
Heavy Chain
37
Light Chain
25
Figure 1 E
75
Heavy Chain
55
CUT
37
TIAR
Figure 1 E
100
75
55
Heavy Chain
37
25
DAZL
Figure 2 B
75
sh1
55
PTEN
PTEN
75
sh2
55
Figure 2 B
75
sh1
55
GFRα1
sh2
75
55
GFRα1
Figure 2 F
sh1
sh2
RPS6
Figure 2 F
sh1
sh2
p-RPS6
Figure 2 B
100
sh1
sh2
75
NEDD4
55
Figure 2 B
sh1
sh2
15
NANOS2
Figure 2 B
sh1
sh2
55
β-TUBULIN
Figure 2 H
Sh1-DMSO
100
NEDD4
75
Figure 2 H
Sh1-4OHT
100
NEDD4
75
Figure 2 H
25
15
NANOS2
Sh1-DMSO
Figure 2 H
25
15
NANOS2
Sh1-4OHT
75
55
β-TUBULIN
Sh1-DMSO
CUT
75
55
β-TUBULIN
Sh1-4OHT
Figure 7 A
100
75
NEDD4
55
Heavy Chain
Figure 7 A
Light Chain
25
15
NANOS2

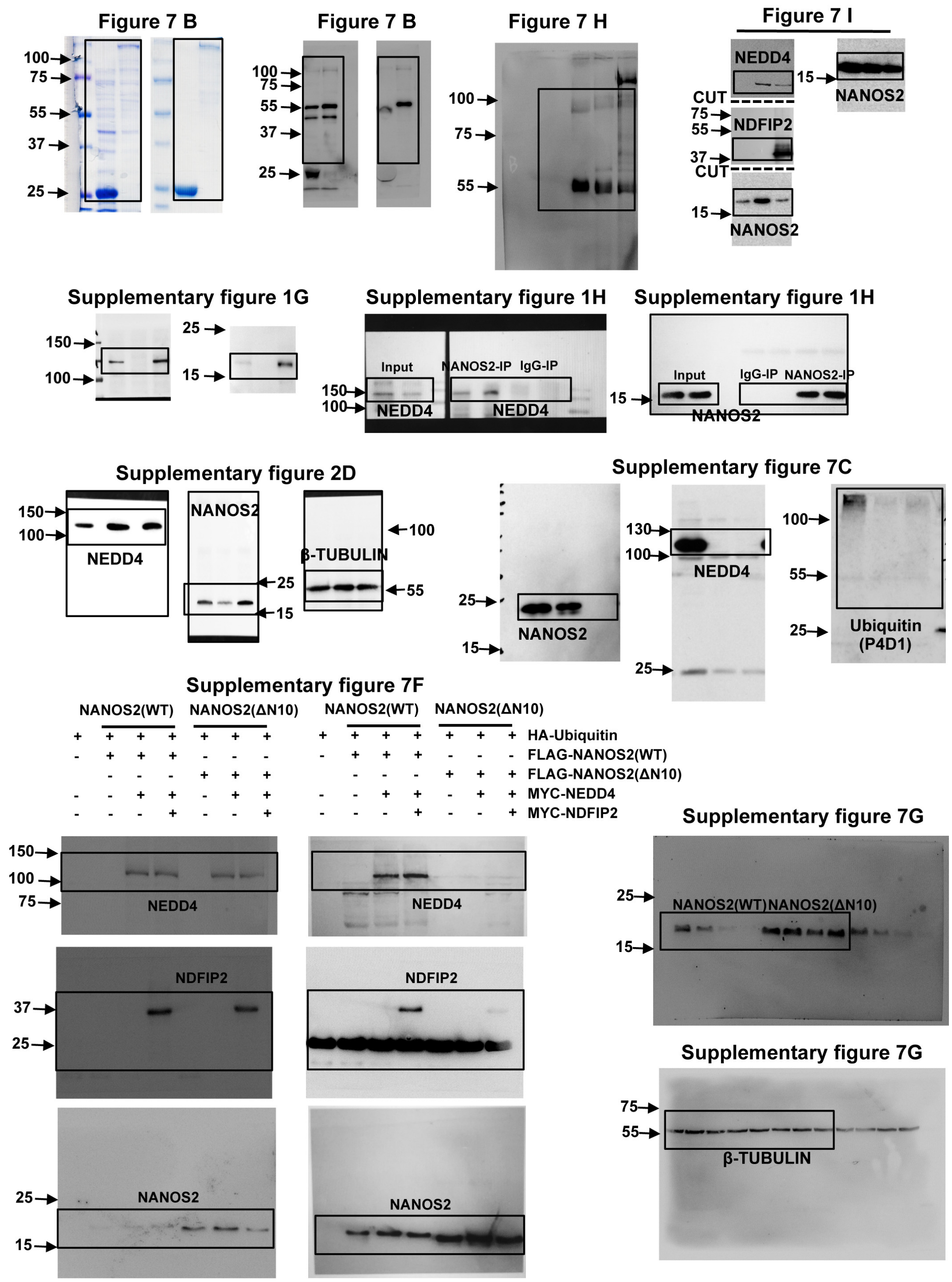

Supplementary figure 8. Uncropped scans of the most important western blots images shown in this paper.

Supplementary Table 1: PCR primers

Gene name		Sequence (5' to 3')
Nanos2	Forward	TCCCATCCTGAGGCACTATGT
	Reverse	ACTGCTGTTGAGTGGACAATAC
Nedd4	Forward	CAGACCAGGCTGAGGAGTTAG
	Reverse	AGGGCTTGGCCTTTTCCACT
C-kit	Forward	GCCACGTCTCAGCCATCTG
	Reverse	GTCGCCAGCTTCAACTATTAACT
GFRα1	Forward	GCACCAAGTACCGCACACT
	Reverse	GCGGCAGTTGTAGAGAGACTTC
plzf	Forward	CTGCGGAAAACGGTTCCTG
	Reverse	GTGCCAGTATGGGTCTGTCT
sohlh1	Forward	AGCCAGACTCCGGTATAGCCA
	Reverse	GGCATTTGAGCACAAGCAGTG
sohlh2	Forward	CTTTGGAGGGAGCAGTGAGAG
	Reverse	GAATAGGGAGTCAGGGGCCA
taf7l	Forward	GACAACGTCTCACTGCCTGC
	Reverse	TACATCTCAAAAGTCACACAAAT

mRNA level of spermatogonial specific genes were quantified by the above primers used in this study.

Supplementary Methods

Antibodies dilution in this study:

Immunofluorescence (IF) and immunohistochemistry (IHC)

rabbit polyclonal anti-NANOS2 (1:200), mouse-monoclonal NANOS2(1:50), polyclonal anti-NEDD4 (Abcam, 1:200), monoclonal anti-TIAR (BD Biosciences, 1:200), monoclonal anti-TfR (Novus Biologicals, 1:200), monoclonal anti-LAMP1 (BD Biosciences, 1:200), polyclonal anti-CDH1(1:200), polyclonal anti-PLZF (Santa Cruz Biotechnology, 1:200), polyclonal anti-EIF4E (Cell Signaling Technology, 1:300), polyclonal anti-SYCP3 (Abcam, 1:500), polyclonal anti-GATA1 (Santa Cruz Biotechnology, 1:200), polyclonal anti-GFRα1 (R&D Systems,1:200), polyclonal anti-DAZL (Abcam, 1:500), polyclonal anti-p-RPS6 (Cell Signaling Technology, 1:300), polyclonal anti-Ki67 (Thermo, 1:300), polyclonal anti-c-PARP (Cell Signaling Technology, 1:300), and monoclonal anti-FLAG (Sigma, M2, 1:1000). Detection of primary antibodies was performed with Alexa 594 or 488 Donkey anti-mouse (Invitrogen, 1:1000), Alexa 488 Donkey anti-Rabbit (Invitrogen, 1:1000), goat anti-Armenian hamster (eBioscience, 1:500), goat anti-Rat Cy5 (Invitrogen, 1:1000), and Donkey anti-goat Cy5 (Invitrogen, 1:1000).

Western blot

polyclonal anti-NANOS2 (1:100), polyclonal anti-NEDD4 (Abcam, 1:1000), polyclonal anti-PABP1 (Abcam, 1:1000), anti-TIAR (BD Biosciences, 1:1000), polyclonal anti-RPS6 or anti-pRPS6 (Cell Signaling Technology, 1:1000), monoclonal anti-TIAR (BD Biosciences, 1:1000), polyclonal anti-DAZL (Abcam, 1:1000), polyclonal anti-GFRα1 (R&D Systems,1:500), monoclonal anti-FLAG (Sigma, 1:5000), polyclonal anti-PTEN (Abcam, 1:500), polyclonal anti-MYC (Sigma, 1:1000), polyclonal anti-ubiquitin (Santa Cruz Biotechnology, 1:200), polyclonal anti-NDFIP2 (Santa Cruz Biotechnology, 1:200), and monoclonal anti-β-TUBULIN (Sigma, 1:2000). Horseradish peroxidase (HRP) conjugated secondary antibodies were from Cell Signaling Technology (1:5000).

Supplementary References

1. Suzuki, A., Igarashi, K., Aisaki, K., Kanno, J. & Saga, Y. NANOS2 interacts with the CCR4-NOT deadenylation complex and leads to suppression of specific RNAs. *Proceedings of the National Academy of Sciences of the United States of America* **107**, 3594-3599 (2010).
2. Suzuki, A., Saba, R., Miyoshi, K., Morita, Y. & Saga, Y. Interaction between NANOS2 and the CCR4-NOT deadenylation complex is essential for male germ cell development in mouse. *PloS one* **7**, e33558 (2012).
3. Zhou, Z. *et al.* RNA Binding Protein Nanos2 Organizes Post-transcriptional Buffering System to Retain Primitive State of Mouse Spermatogonial Stem Cells. *Developmental cell* **34**, 96-107 (2015).
4. Hasegawa, K., Namekawa, S.H. & Saga, Y. MEK/ERK signaling directly and indirectly contributes to the cyclical self-renewal of spermatogonial stem cells. *Stem cells* **31**, 2517-2527 (2013).
5. Kanatsu-Shinohara, M. *et al.* Long-term proliferation in culture and germline transmission of mouse male germline stem cells. *Biology of reproduction* **69**, 612-616 (2003).
6. Sada, A., Hasegawa, K., Pin, P.H. & Saga, Y. NANOS2 acts downstream of glial cell line-derived neurotrophic factor signaling to suppress differentiation of spermatogonial stem cells. *Stem cells* **30**, 280-291 (2012).
